# A cell-based fluorescent system and statistical framework to detect meiosis-like induction in plants

**DOI:** 10.3389/fpls.2024.1386274

**Published:** 2024-07-08

**Authors:** Tanner M. Cook, Eva Biswas, Siddique I. Aboobucker, Somak Dutta, Thomas Lübberstedt

**Affiliations:** ^1^ Iowa State University, Department of Agronomy, Ames, Iowa, IA, United States; ^2^ Iowa State University, Department of Statistics, Ames, Iowa, IA, United States

**Keywords:** high-throughput, single-cell analysis, plant breeding, *in vitro* biology, meiosis-like induction, protoplasts

## Abstract

Genetic gains made by plant breeders are limited by generational cycling rates and flowering time. Several efforts have been made to reduce the time to switch from vegetative to reproductive stages in plants, but these solutions are usually species-specific and require flowering. The concept of *in vitro* nurseries is that somatic plant cells can be induced to form haploid cells that have undergone recombination (creating artificial gametes), which can then be used for cell fusion to enable breeding in a Petri dish. The induction of *in vitro* meiosis, however, is the largest current bottleneck to *in vitro* nurseries. To help overcome this, we previously described a high-throughput, bi-fluorescent, single cell system in *Arabidopsis thaliana*, which can be used to test the meiosis-like induction capabilities of candidate factors. In this present work, we validated the system using robust datasets (>4M datapoints) from extensive simulated meiosis induction tests. Additionally, we determined false-detection rates of the fluorescent cells used in this system as well as the ideal tissue source for factor testing.

## 1 Introduction

Advances in cultivar development have enabled plant breeders to create crops that have higher yields, resistance to environmental and biological stresses, nutrient fortification, and decreased nutrient requirements. Breeding, however, requires gamete formation, which occurs during flowering, enabling genetic mating to develop new varieties and cultivars. Time to flowering and gamete formation may take months such as in maize (De La Fuente et al., 2013), or decades, as is the case in some woody species (Hackett, 1985). Speed breeding (Watson et al., 2018; Jähne et al., 2020), phytohormonal induction of early flowering (Espinosa et al., 2017), and dwarf plants (Bugbee et al., 1999; McCaw et al., 2016; Li et al., 2017, Li et al., 2018) are breeding technologies that have focused on reducing the time from seed-to-seed. Speed-breeding has probably had the greatest impact on reducing breeding cycle time as it substantially decreases flowering time by capitalizing on manipulations of growth conditions (Ghosh et al., 2018; Watson et al., 2018; Pandey et al., 2022; Rodrmguez et al., 2023). These breeding advances still require mature plants with floral organs for gamete development, however, and are species-specific. Alternatively, *in vitro* breeding programs have been suggested as a way to allow meiosis to occur independent of gamete formation during flowering (De La Fuente et al., 2013; Murray et al., 2013).

Meiosis is central to plant breeding, as it generates novel allelic combinations in recombinant offspring, which can subsequently be subjected to selection. DNA replication followed by recombination and two cell divisions leads to gamete formation. These gametes can then be fused to make new genotypes. Meiotic underpinnings have been investigated in numerous studies in the past (reviewed by Cook et al., 2023), but factors leading to efficient *in vitro* meiosis induction in plants are yet to be identified. In a mammalian system, *in vitro* meiosis has been accomplished at rates around 1% (Medrano et al., 2016). Additionally, previous studies with plants have identified factors that reduce chromosomes to a haploid state *in vitro* in a meiosis-like fashion (Yoshida and Yamaguchi, 1973; Chen et al., 2000; Yihua et al., 2001), however, the efficiency at which this is done is unknown (Yan et al., 2017). Thus far, *in vitro* nurseries (IVN; De La Fuente et al., 2013; Cook et al., 2023) or cycling of gametes *in vitro* (COGIV; Murray et al., 2013), are only concepts. If we can learn how to trigger meiosis induction *in vitro*, we are closer to making these ideas a reality.

Previously, we published an article describing the methodology to develop a system to test various factors for their meiosis induction capabilities (Cook et al., 2024). This current article builds on our previous work as we use a robust set of data to validate the system while providing insight into the development of this tool. Herein, we ran extensive simulated meiosis induction tests (uni-fluorescent cells being spiked-in to a population of bi-fluorescent cells) to determine the sensitivity and statistical limits of our system with more than 1.1 million events analyzed (over 6 million data points) and multiple biological replicates. Further, we have determined false-detection rates of our fluorescent genotypes and the best tissue source for testing. We have also used the dataset presented in this article and the previously discussed statistical model to determine recovery rates of uni-fluorescent red-expressing cells within bi-fluorescent cell populations. Thus, the validation of this tool provides evidence that our system can be put into service for accurate testing of a candidate’s ability to induce a meiosis-like state *in vitro*.

Specifically, this article assesses (i) the establishment of fluorescent *Arabidopsis thaliana* lines that can be sexually crossed to generate bi-fluorescent cells for evaluating meiosis-like induction and (ii) spike-in pilot experiments to determine the sensitivity and statistical limits of detecting uni-fluorescent cells in large populations of bi-fluorescent cells.

## 2 Materials and methods

### 2.1 Rationale

#### 2.1.1 Experiment 1: Bi-fluorescent genotype development and fluorescent tissue screening

For the development of a high-throughput meiosis-like induction detection system, we needed two different fluorescent markers with high fluorescence expression that were far enough apart on the electromagnetic spectrum that they could be easily differentiated. We tested and characterized 25 fluorescent *Arabidopsis* lines with red or green fluorescence. Seed was obtained from publicly available mutant stock resources, Iowa State University researchers, or developed using *Agrobacterium*-mediated transformation (**Supplementary Table S1**). We observed the roots under a compound fluorescent microscope to qualitatively assess fluorescence. During selection, important considerations were strong fluorescence and single-locus insertions. After confirmation of single-locus insertion and fluorescence, we isolated leaf and/or root protoplasts from promising lines for analysis with flow cytometry. The necessity for single-locus marker genes was that with two or more genetic loci there is a reduction of power in haploid cell detection (i.e., discrimination from diploid cells). Constitutive expression was an important consideration to maximize the chance that if we were successful in inducing *in vitro* meiosis, the cells would still fluoresce. We screened lines that used either a ubiquitin or a CaMV 35S promoter. Due to the ease of isolation (Sheen, 2001; Davey et al., 2005; Yoo et al., 2007; Chupeau et al., 2013; Eeckhaut et al., 2013; Nelms and Walbot, 2019), we decided that protoplasts were the best choice for simulated high-throughput meiosis induction evaluation. However, protoplasts are fragile and susceptible to environmental changes, thus we decided that factors potentially inducing meiosis would need to be evaluated in the future on dividing cells *in vitro* before protoplasts were isolated to assess the percentage of haploid cells via flow-cytometry. Multiple tissues were analyzed for red fluorescent protein (RFP) and green fluorescent protein (GFP) expression and assessed using pairwise comparisons of cells with leaf, root, and callus tissue sources.

#### 2.1.2 Experiment 2: Fluorescent misclassification rates of callus-derived protoplasts

Once high-expressing RFP and GFP fluorescent lines were identified, we characterized fluorescent callus protoplasts for false-negative and false-positive rates based on cell classifications using fluorescent thresholds (**Supplementary Table S2**) into RFP, GFP, GFP/RFP, and non-fluorescing cells. Misclassified cells are present in biological systems, and it is important to understand the level of misclassification for cells derived from known genotypes. This is a necessary prerequisite to develop tests capable of detecting rare events of meiosis induction. A significant increase of cells classified as RFP or GFP compared to the control (not treated for meiosis induction) will be indicative of haploid cells. In this present study, protoplasts isolated from callus were assessed for FITCA (GFP expression) and PECF594A (RFP expression) fluorescence for all samples after quality gating to determine misclassification rates. Setting consistent thresholds allowed us to make comparisons across genotypes from different isolation dates (**Supplementary Table S2**).

#### 2.1.3 Experiment 3: Detection of simulated meiosis induction and statistical analysis

Assessing the sensitivity of our single-cell system was critical in determining what rates of simulated meiosis-like induction could be detected across multiple samples and treatments. We conducted spike-in analyses, in which known concentrations of cells from the RFP line were mixed with known concentrations of cells from the GFP/RFP genotype. These assays were conducted to obtain actual classification rates in simulated meiosis tests. Haploid cells were used in multiple spike-in tests from two different biological sources (**Supplementary Figure S1**). Haploid cells were used to provide material as similar to artificial gametes as possible. Spike-in experiments were also conducted with diploid cells from the RFP line.

### 2.2 Plasmid design and Arabidopsis transformation

We obtained the plasmid pGFPGUSPlus containing eGFP (Vickers et al., 2007) from Addgene (Plasmid #64401). *XbaI* and *SacI* sequences were added by PCR amplification to the eGFP coding region using eGFP-Fw-XbaI and eGFP-Rv-SacI primers. The amplicon was digested with *XbaI* and *SacI* and the resulting fragment was ligated to the pTF101 vector under the control of the 35S promoter as previously described (Aboobucker et al., 2021). The new plasmid is named “pTF101–35S-eGFP” and the vector contains a bar-selectable marker under the 35S promoter. mRFP1 coding sequence was amplified from pRU1144 plasmid (Addgene plasmid #14474; Karunakaran et al., 2005) with *BamHI* and *SacI* sites using primers mRFP1-Fw-BamHI and mRFP1-Rv-SacI. Sub-cloning mRFP1 in the pTF101 vector was similar to the process described for eGFP, which resulted in “pTF101–35S-mRFP1”. Selected clones were verified by Sanger sequencing at the ISU DNA Facility. Plasmids were introduced into *Agrobacterium tumefaciens* strain C58C1 by the freeze-thaw method (Holsters et al., 1978). For *Arabidopsis* transformation, the floral dip method was used (Clough and Bent, 1998). Putative transformants were those resistant to 60 mg/L glufosinate ammonium sprayed on seedlings seven days after sowing. Further molecular confirmation for eGFP, mRFP1, and bar sequences was carried out on DNA isolated from the putative transformants.

### 2.3 Plant cultivation, crossing, and callus induction

*Arabidopsis thaliana* Columbia ecotype (CS70000) used as genetic background, was obtained from the Arabidopsis Biological Resource Center. Transgenic seeds were sterilized, planted, sexually crossed, and callus was initiated and subcultured, all of which is described in Cook et al. (2024). Seeds were stratified for four to eight days.

### 2.4 Protoplast isolation and spike-in testing

Protoplasts for spike-in experiments were isolated from callus tissue that was subcultured up to 39 days before isolation, using the method outlined previously (Cook et al., 2024). A 10µL protoplast sample was placed on a Marienfeld 0.0025mm^2^ hemacytometer to estimate the cell concentration. The hemacytometer measurement was repeated multiple times for each sample and the average was used to determine the protoplast concentration.

For the spike-in sample preparations, known concentrations of cells from the RFP line were mixed with a known concentration of cells from the GFP/RFP genotype and mixed thoroughly. Total concentrations of RFP were 0.74%, 1%, 1.5%, and 5%.

### 2.5 Flow cytometry

Protoplasts were isolated and analyzed as described previously (Cook et al., 2024). Callus-derived protoplasts were analyzed using consistent settings for six different channels (**Supplementary Table S2**).

### 2.6 Root imaging specifications

For root imaging, we used a fluorescent Olympus SZH10 stereo microscope equipped with a camera and Leica software. A Texas red filter (excitation: 562/40, emission: 624/40) and an L5 filter (excitation: 480/40, 527/30) was used for visualization.

### 2.7 Statistical considerations

The statistical model and estimation method for estimating RFP rates after accounting for misclassifications by the gating scheme are published in more detail in a previous article from our group (Cook et al., 2024). We perform a simple linear regression analysis to investigate the relationship between the estimated and the true spike-in percentages and how closely the estimated spike-ins mimic the true value of spike-ins. To that end, let, *s_il_ and* ${\hat{s}}_{il}$ be the true and estimated $l^{th}$ spike-in rates in the $i^{th}$ set of experiments. We use the simple linear regression model:


$${\hat{s}}_{il}=\beta_{o}+\beta_{1}s_{il}+e_{il}$$

for i=1,…,K  and $l=3,\ldots ,L_{i}$ , with $e_{il}$ being normal random error for each observation. Here, under lossless detection of spike-in rates $\beta_{1}$ should be 1. However, in practice we expect $\beta_{1}$ to be less than 1 because some cells are destroyed in the process.

## 3 Results

### 3.1 Experiment 1: Bi-fluorescent genotype development and fluorescent tissue screening

**Figure 1:**
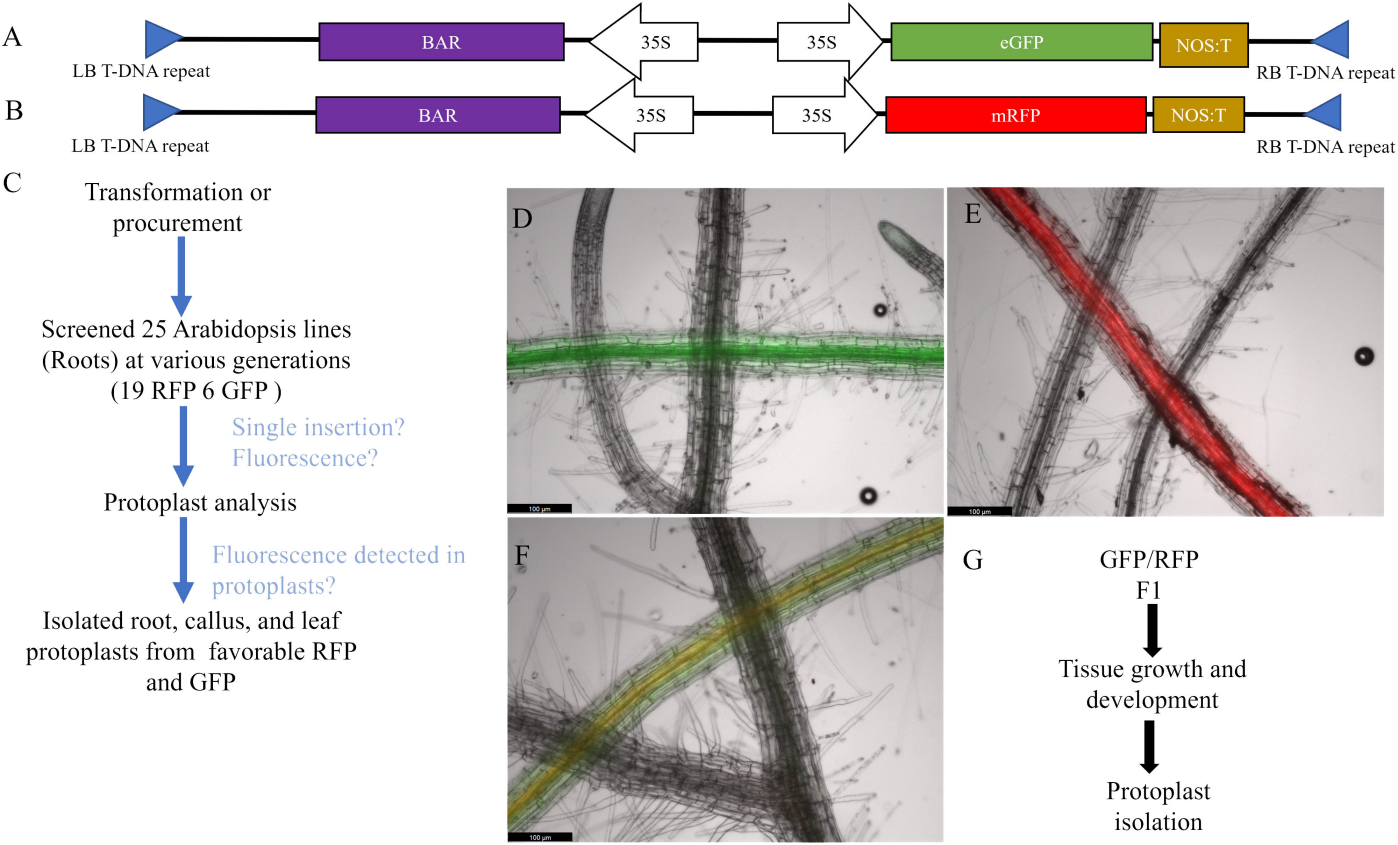
Bi-fluorescent genotype development and screening. **(A, B)** Diagram of the TDNA used to overexpress fluorescent genes in *Arabidopsis*. Both RFP and GFP are driven by a constitutive 35S promoter and include a constitutively expressed BAR gene for selection. **(C)** *Arabidopsis* roots were screened under a compound fluorescent microscope and assessed for single insertions. Candidate fluorescent lines were then isolated for protoplasts and analyzed using flow cytometry. Favorable lines were used to isolate leaf, root, and callus protoplasts for further analysis. **(D–F)** Fluorescent protein expression in roots with WT root crossed over in the same image. Scale bar = 100µm. **(D)** GFP expressing *Arabidopsis* root. GFP fluorescence was captured with 900ms of exposure and the image brightness was set at 25. **(E)** RFP expressing *Arabidopsis* root. RFP fluorescence was taken with 500ms of exposure with brightness set at 50. **(F)** GFP/RFP expressing *Arabidopsis* root. GFP and RFP fluorescence were captured with the same specifications for the respective fluorescent marker. All images have GFP, RFP, and brightfield overlayed. **(G)** Schematics workflow of protoplast isolation from callus derived from the GFP/RFP *Arabidopsis* genotype.

*Two Agrobacterium-*mediated transgenic lines were identified and chosen for further development of a high-throughput *in vitro* system for meiosis induction detection. **Supplementary Figures S2**, **S3** identify two RFP and two GFP-expressing *Arabidopsis* lines analyzed for phenotypic analysis. We selected 35s::RFP 2–3 and 35s::GFP 1–3 lines (now referred to as RFP and GFP, respectively) for additional use, based on the fact these lines displayed high fluorescence expression in roots (**Figure 1**). Moreover, more than 50% of the protoplasts generated from the leaves of these lines showed expression of the fluorescent markers (**Supplementary Figures S1**, **S3**). To ensure that the transgenes conferring either RFP or GFP were located at a single locus, the inheritance of a linked BAR-resistance gene was evaluated among the selfed progeny from a heterozygous parent. The data presented in **Supplementary Table S3** are consistent with the RFP and GFP transgenes being at a single genetic locus.

**Figure 2:**
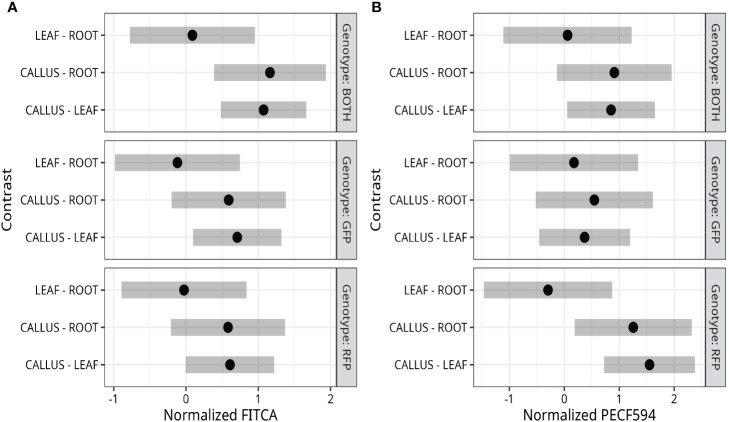
Fluorescence detection in callus, leaf, and root protoplasts. **(A, B)** Tukey adjusted 95% familywise confidence intervals of pairwise differences between protoplasts derived from callus, leaf, and root. Data were normalized using orderNorm (Peterson, 2021). The median normalized fluorescence of cells was calculated for each replicate and analyzed through a two-way model with genotype and tissue main effects and interactions.

Bi-fluorescent plants were generated by crossing the RFP line and the GFP line (**Figure 1F**). Subsequently, fluorescence was evaluated in protoplasts isolated from roots, leaves, and callus cultures from these F1 plants and their parental lines. Pairwise comparisons of the normalized FITCA (GFP expression) and PECF594A (RFP expression) fluorescence intensity values are presented in **Figure 2**. There was evidence for a higher GFP (FITCA) fluorescence in callus-derived protoplasts than in leaf protoplasts isolated from the GFP/RFP and GFP genotypes. These callus protoplasts also showed higher GFP (FITCA) fluorescence than root protoplasts from the GFP/RFP genotype (**Figure 2A**). Similarly, RFP (PECF594A) fluorescence was higher in callus-derived protoplasts as compared to leaf protoplasts from the GFP/RFP genotype and RFP line (**Figure 2B**). Furthermore, RFP (PECF594A) fluorescence was also higher in callus protoplasts than in root-derived protoplasts from the RFP line (**Figure 2B**).

### 3.2 Experiment 2: Fluorescent misclassification rates of callus-derived protoplasts

Thresholding was used to classify callus-derived protoplasts from the GFP, RFP, and GFP/RFP genotypes. Fluorescence detection rates for each genotype, as classified by thresholding, are presented in **Table 1**. Callus-derived protoplasts from the RFP line were correctly identified 66.5% of the time while those isolated from the GFP line were correctly identified 40.3%. The majority of misclassifications were identified as wild-type or non-fluorescing cells. Callus-derived protoplasts isolated from the GFP/RFP genotype were correctly identified 11.8% of the time while GFP false-identification rates exceeded 55% and RFP false-identification was less than 0.1%. Additionally, when callus-derived protoplasts from the RFP line were analyzed, cells that were falsely detected as being GFP or GFP/RFP were at rates below 0.1%. When callus-derived protoplasts from the GFP line were analyzed, cells that were falsely detected as being RFP or GFP/RFP were detected at rates below 3.5%.

**Table 1:** Cell classifications of collated callus protoplasts defined from gating which were isolated from the GFP, RFP, and, GFP/RFP genotypes.

Classification	Collated GFP	%	Classification	Collated RFP	%	Classification	Collated GFP/RFP	%
GFP	88619	40.33%	GFP	0	0.00%	GFP	146951	55.04%
WT	123903	56.39%	WT	49400	33.50%	WT	88435	33.12%
RFP	55	0.03%	RFP	97980	66.45%	RFP	98	0.04%
Both	7151	3.25%	Both	70	0.05%	Both	31527	11.81%
Total	219728		Total	147450		Total	267011	

### 3.3 Experiment 3: Detection of simulated meiosis and statistical analysis

**Figure 3:**
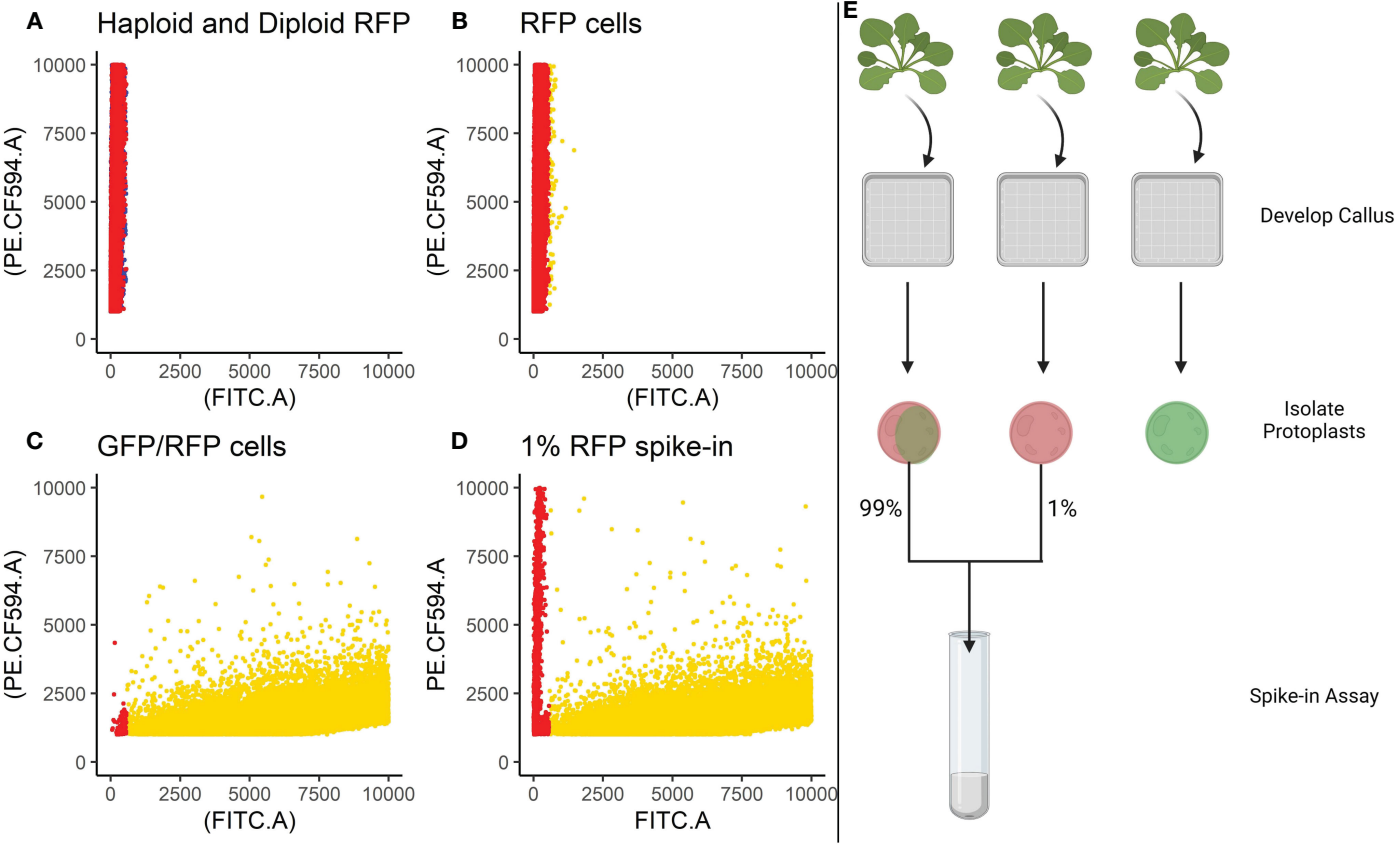
Spike-in experiments. Cells isolated from the RFP line were spiked into a tube containing cells isolated from the GFP/RFP genotype at a known concentration of 1%. **(A)** Provides the overlapping population distributions for the haploid cells (blue) and diploid cells (red) isolated from the RFP line. **(B)** Classification of cells isolated from the RFP line. **(C)** Classification of F1 cells isolated from the GFP/RFP genotype. **(D)**. Classification of spike-in tests containing 1% cells from the RFP line and 99% cells from the GFP/RFP genotype. **(B–D)** Gold points represent cells classified as GFP/RFP. Red points represent cells classified as RFP. B and C are collations of the samples used to create the 1% spike-ins. Cells were classified using a gating classification system shown in **Supplementary Table S2**, cells classified as GFP and WT were excluded. Cells from multiple samples were collated together for the graphs. **(E)** Provides a visual summary of the spike-in assay setup. Created with BioRender.

**Figure 4:**
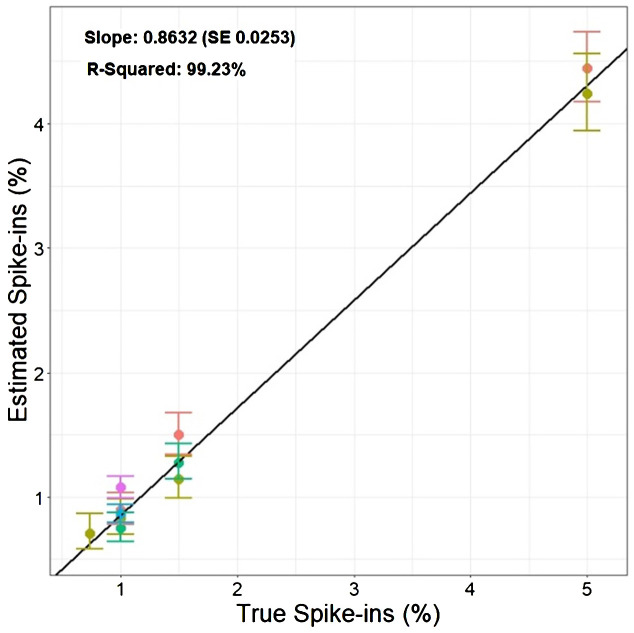
Estimated percentage of cells classified as RFP vs. True RFP spike-in percentages.: The graph compares the known percentages of RFP cells added to the GFP/RFP cell population against the estimated percentage derived from the multinomial statistical model. The solid points show the estimated spike-in levels obtained from multiple experiments performed on different dates denoted by different colors. The vertical bars present the 95% confidence intervals associated with each estimate of spike-ins. The black solid line represents the regression line obtained by fitting the estimated spike-ins on the true spike-ins level.

Based on the findings in Experiment 2, we decided to focus on RFP protoplasts, as they could be much better discriminated from the protoplasts classified as GFP/RFP as compared to what GFP protoplasts could. Because of the inability to differentiate cells classified as GFP and those classified as GFP/RFP; GFP, and GFP/RFP cell classifications were combined in a new merged class for a multinomial statistical model (see materials and methods). To determine if minute changes in fluorescent cell population proportions could be detected even with the inability to separate GFP and GFP/RFP classified cells, callus-derived protoplasts from the GFP/RFP genotype (**Figure 1F** and **Table 1**) were spiked with known amounts of callus-derived protoplasts from the RFP line (**Figure 1E** and **Table 1**), both haploid and diploid, and detected via flow cytometry (**Figure 3**). **Figure 3A** provides the overlapping population distributions for the haploid and diploid cells isolated from the RFP line. **Figure 3D** provides an overlapping population distribution for a collation of individual 1% RFP spike-in experiments that were developed from cells contained in **Figures 3B, C**. Classification rates are shown in **Table 2** and **Figure 4**. The estimated spike-in rates, from the multinomial model analysis, are plotted against the true spike-in rates in **Figure 4**. Collated classification rates from multiple spike-in experiments detected mean values of 0.88%, 1.3%, and 4.3% for 1%, 1.5%, and 5% spike-ins, respectively (**Table 2**), and the confidence interval for the spike-in rate of 0.74% is (0.58%, 0.86%). Thus, these data indicate that spike-in percentages as low as 0.74% and as large as 5% can accurately be detected with this biological system and statistical model. The recovery rate of RFP cells mixed into GFP/RFP cells was 86% (**Figure 4**: βˆ 1 = 0.86, SE = 0.025, R2 = 99.2%, p-value< 0.0001). The inability to differentiate GFP and GFP/RFP classified cell populations decreases the detection sensitivity but given the large number of cells capable of being analyzed by flow cytometry and the low misclassification rates of fluorescent cells with the RFP line, detection of small changes in fluorescent cell populations can be detected when observing RFP using our current system.

**Table 2:** Results of individual spike-in experiments.

Date of Isolation	Total Fluorescing cells of spike-in	Spike-in percent expected	Spike-in percent estimated (95% CI)	Haploid or Diploid RFP Cells	RFP2–3 Plant	GFP1–3/RFP2–3 F1 Plant
220804	15,795	0.74	0.7 (0.58, 0.86)	Haploid	R3	GxR10
220713	17,695	1.00%	0.89 (0.77, 1.03)	Haploid	R3	GxR10
220804	17,899	1.00%	0.82(0.69,0.98)	Haploid	R3	GxR10
221101	19,825	1.00%	0.74 (0.63, 0.87)	Haploid	R11	GxR2
230125	62,133	1.00%	0.87 (0.79, 0.94)	Diploid	R20	GxR150
230125	59,610	1.00%	1.08 (0.99, 1.16)	Diploid	R21	GxR151
Collated	177,162	1.00%	0.88	Collated	Collated	Collated
220713	17,425	1.50%	1.5 (1.34, 1.67)	Haploid	R3	GxR10
220804	17,786	1.50%	1.14 (0.99, 1.32)	Haploid	R3	GxR10
221101	18,756	1.50%	1.27 (1.14,1.43)	Haploid	R11	GxR2
Collated	53,967	1.50%	1.3	Collated	Collated	Collated
220713	17,570	5.00%	4.45 (4.18,4.73)	Haploid	R3	GxR10
220804	17,372	5.00%	4.24 (3.94, 4.56)	Haploid	R3	GxR10
Collated	34,942	5.00%	4.35	Collated	Collated	Collated

Summaries of individual spike-in experiments where multiple tests were conducted. The results show the percentage of RFP cells estimated out of fluorescent cells in each sample along with 95% confidence intervals (see Statistical Considerations). Both haploid and diploid cells were tested in the 1% spike-in experiments.

## 4 Discussion

### 4.1 Bi-fluorescent genotype development to detect simulated meiosis-like induction

We developed an *Arabidopsis thaliana* bi-fluorescent F1 genotype to create a single-cell meiosis-like induction evaluation system using flow cytometry. We validated our system with spike-in experiments using flow cytometry and a multinomial statistical model. Borges et al. (2012) used a similar bi-fluorescent setup to purify sperm cells and vegetative nuclei using fluorescence-activated cell sorting (FACS) based on the presence of mRFP and eGFP. In our study, fluorescent genotypes underwent callus induction to obtain rapidly dividing cells that can easily be isolated as protoplasts. Thresholding enabled the classification of RFP, GFP, and GFP/RFP cell populations using red and green fluorescence properties. Spike-in experiments analyzed with a multinomial model allowed for the detection of uni-fluorescent cells added to bi-fluorescent cells at rates as low as 1%. Further, these spike-in experiments helped to develop and test a statistical framework for future screening efforts of factors triggering meiosis-like induction. Based on our tests, to detect cell population changes of 0.25% (a 1% meiosis induction rate with the current system), it is necessary to have a sample size of around 200,000 cells (for 80% power), which is still within the capabilities of flow cytometry. Further, the recovery rate is predicted to be 0.2399% with SE 0.05610%.

### 4.2 Misclassification and fluorescence expression considerations

**Figure 5:**
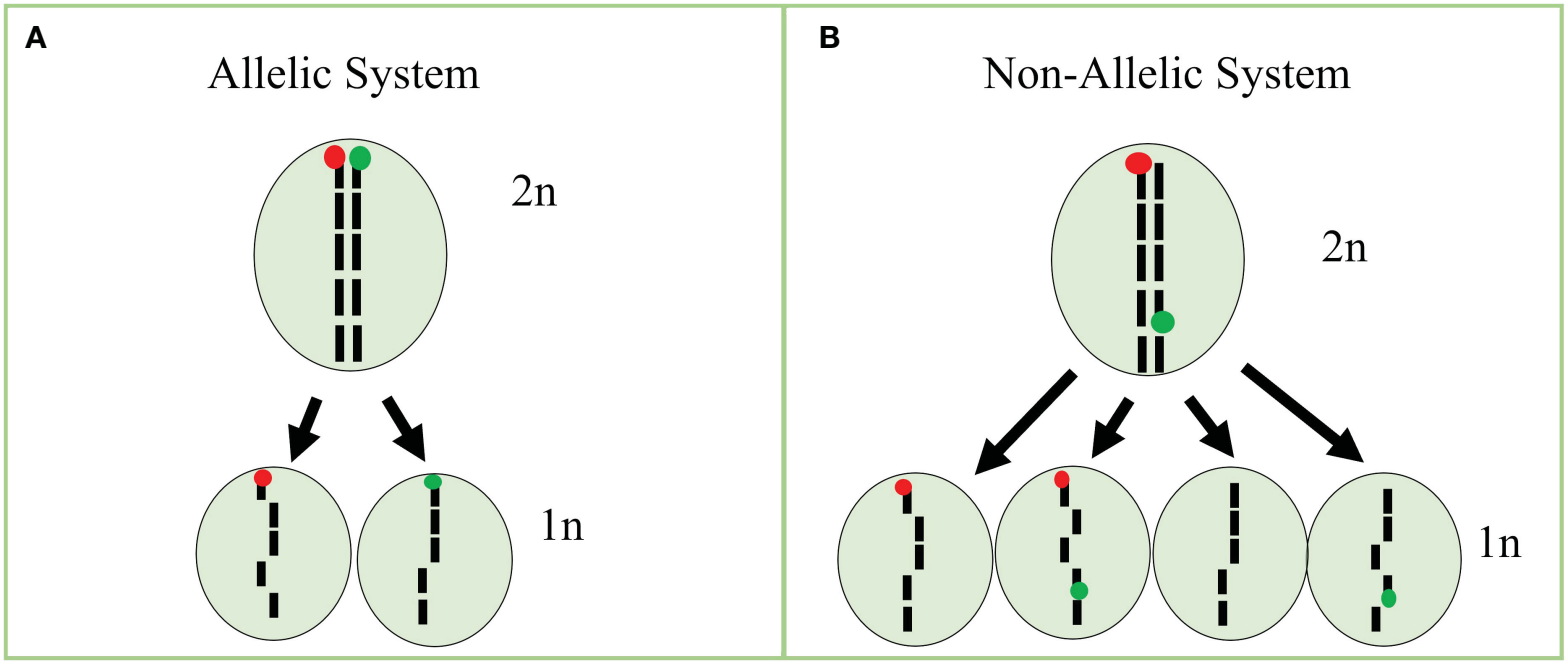
Bi-fluorescent allelic and non-allelic systems using single cells. **(A)** Treatment of a diploid (2n) cell carrying bifluorescent markers in an allelic system would result in haploid (1n) cells with only RFP or GFP if meiosis-like induction occurred. **(B) **Treatment of a diploid (2n) cell carrying bifluorescent markers in the non-allelic system would result in haploid (1n) cells with RFP, GFP, GFP/RFP, or no expression if meiosis-like induction occurred. Figure based on our previous work (Cook et al., 2023).

In our tests, none of the detection rates of fluorophores in cells reached 100%. For instance, protoplasts isolated from our GFP line had cells classified as a majority GFP and WT, whereas a minority of cells were classified as RFP and GFP/RFP. We also observed that RFP fluorescence is reduced in our GFP/RFP genotype. As a consequence, data from the GFP and GFP/RFP cells overlapped substantially and could not be accurately differentiated. The overlapping cell populations resulted in a high-false positive rate for cells classified as GFP in the GFP/RFP genotype. Because of this, we combined the cell classes of GFP and GFP/RFP into one class for statistical analysis (See Cook et al., 2024). The decreased RFP expression in the GFP/RFP genotype can potentially be explained by mutations, gene silencing due to two TDNA constructs being present (Matzke et al., 1989), and homology with multiple 35S promoters (Daxinger et al., 2008; Mlotshwa et al., 2010). High transcript numbers due to multiple gene copies led to silencing as well in previous studies (Schubert et al., 2004), but we are uncertain this is a major factor in our system as there were only two copies of the BAR resistance gene, one copy of RFP and one copy of GFP in our GFP/RFP plants. Schubert et al. (2004) found that six copies of GFP under the 35S promoter led to silencing while silencing was seen with only three copies of GUS. Further testing will be required to fully understand the expression characteristics in our samples. In contrast to the overlapping GFP and GFP/RFP classified cells, RFP cells were easily separated from GFP/RFP cells. We were able to efficiently detect low percentages of RFP protoplasts when observing RFP-only cells in our spike-in tests. Since induced haploid cells are expected in equal numbers of RFP and GFP-expressing cells, our assay can be efficiently used by focusing on the detection of cells classified as RFP. Potential improvements to increase the efficiency of our assay include: (i) using different promoters to decrease the risk of homology-mediated silencing, (ii) increasing fluorescence expression with more efficient RFP proteins, and (iii) development of allelic fluorescent markers. As similarities in promoters derived from viruses can result in gene silencing (Daxinger et al., 2008; Mlotshwa et al., 2010), increasing the diversity of constitutive promoters will help to mitigate the risk of silencing due to homology. Promoters such as the nopaline synthase and ubiquitin promoters may be potential candidates, especially for resistance genes. Further, constructs without resistance genes may be an option as genotyping can take place instead, thus eliminating the need for multiple constitutive promoters. A more effective red fluorophore such as mCherry, which has brighter fluorescence and is more photostable than mRFP1 (Shaner et al., 2004; Fink et al., 2010), may help to increase the fluorescence intensity when the GFP construct is present in the same cell. Using allelic markers would also substantially increase the efficiency of the current assay (Cook et al., 2023; **Figure 5**). As shown above, uni-fluorescent cells can successfully be identified with RFP at low rates in the system described here, but the efficiency would be doubled with an allelic approach in the current system and quadrupled if GFP-expressing cells could be identified apart from the GFP/RFP cells (**Figure 5**; Equation 1, Equation 2, Equation 3). Efficiency improvements would increase detection limits while decreasing the needed sample size. Using a binomial formula (Lübberstedt and Frei, 2012), efficiency increases using non-allelic and allelic single-cell systems can be estimated. For example, assuming a 100% identification rate, we have calculated the different efficiencies below.

Detecting a simulated 1% meiosis-like induction rate using the current non-allelic system:


(1) $$\;\frac{ln\left(1-0.95\right)}{ln\left(1-0.0025\right)}\;=\;\sim 1,197\;cells\;are\;needed$$

Detecting a simulated 1% meiosis-like induction rate using an allelic system looking only at RFP:


(2) $$\frac{ln\left(1-0.95\right)}{ln\left(1-0.005\right)}\;=\;\sim 598\;cells\;are\;needed$$

Detecting a simulated 1% meiosis-like induction rate using an allelic system looking at RFP and GFP:


(3) $$\frac{ln\left(1-0.95\right)}{ln\left(1-0.01\right)}\;=\;\sim 298\;cells\;are\;needed$$

### 4.3 Detecting low rates of simulated meiosis-like induction in populations of predominantly diploid cells

We were able to detect spike-ins of 1% uni-fluorescent cells in our current non-allelic system (which reflects a meiosis induction rate of 4%). To improve the detection capabilities of the system below 1%, it is necessary to (i) increase the sample sizes, or (ii) minimize misclassification rates. While *in vitro* meiosis induction rates are unknown in plants, we believe it is necessary to accurately detect haploid cells well below 1% due to the work of Medrano et al. (2016).

### 4.4 Screening tool for *in vitro* meiosis-like induction

Overcoming the *in vitro* meiosis induction bottleneck will allow advanced plant breeding ideas such as the IVN (De La Fuente et al., 2013) or the COGIV method (Murray et al., 2013) to be further explored. The biological materials and statistical framework validated in this study provide a scalable system capable of detecting meiosis-like induction in callus cells using fluorescent markers. With this investigation, we provide a resource to researchers to test large numbers of meiosis induction candidates quantitatively and efficiently. Callus was selected as the tissue of choice because of the high rates of cell division, fluorescent expression compared to other tissues (**Figure 2**), and the ability to survive months with proper care. We did not consider cell types such as microspores as they require a plant in reproductive phases which would still present the bottleneck of timing similar to flowering, we tested vegetative tissues (leaf and root) and callus derived from leaf. Using the current callus based system,several induction tests could be run in a short time using this method as multiple samples can easily be analyzed, especially with flow cytometers designed to use 96-well plates. Genetic treatments may also be possible through transformation with *Agrobacterium* co-cultivation or transient transfection, but there are many challenges associated with genetic testing using our system that further feasibility assessments need to take place. While this system provides an efficient and quantifiable method to assess a factor’s ability to induce a meiosis-like fate, further testing of promising candidates will need to take place to ensure that recombination has occurred.

### 4.5 Applications to crop systems

The current system provides an important resource to initially screen a multitude of factors for their ability to induce meiosis. Successful factors can then be used to develop a library that will be tested in crop species. The use of *Arabidopsis* provides a model organism with vast genetic resources and short generational cycles. Indeed, however, there will most likely be changes to dosage concentrations when testing factors in other crop species, but the conservation of meiotic factors across plants provides evidence that a successful factor in one species has promise in another (see Mieulet et al., 2016). The *in vitro* induction of meiosis and future utilization in crop breeding is a difficult road, what we provide here is the first step to enable a systematic approach for step changes in breeding.

## Data availability statement

The data presented in the study and script files are published at https://doi.org/10.25380/iastate.25215446.v1.

## Author contributions

TC: Conceptualization, Formal analysis, Investigation, Validation, Writing – original draft, Writing – review & editing. EB: Formal analysis, Validation, Visualization, Writing – original draft, Writing – review & editing. SA: Conceptualization, Resources, Writing – original draft, Writing – review & editing. SD: Formal analysis, Funding acquisition, Validation, Writing – original draft, Writing – review & editing. TL: Conceptualization, Funding acquisition, Project administration, Supervision, Writing – original draft, Writing – review & editing.

## Supplementary material

Supplementary Table 1 Fluorescent *Arabidopsis* lines screened.

Supplementary Table 2 Gates used for protoplast classification for RFP, GFP, both, and WT classifications.

Supplementary Table 3 Chi-squared results for determining if RFP and GFP are present in single-loci.

Supplementary Figure 1 Plant phenotyping and flow cytometry data for haploid or diploid determination.

Supplementary Figure 2 Root and protoplast screening results of two high-expressing RFP lines.

Supplementary Figure 3 Root and protoplast screening results of two high-expressing GFP lines.

## References

[B1] Aboobucker S. I. Showman L. J. Lübberstedt T. Suza W. P. (2021). Maize zmcyp710a8 mutant as a tool to decipher the function of stigmasterol in plant metabolism. Front. Plant Sci. 12. doi: 10.3389/fpls.2021.732216 PMC8597121 34804084

[B2] Borges F. Gardner R. Lopes T. Calarco J. P. Boavida L. C. Slotkin R. K.. (2012). FACS-based purification of Arabidopsis microspores, sperm cells and vegetative nuclei. Plant Methods 8, 44. doi: 10.1186/1746-4811-8-44 23075219 PMC3502443

[B3] Bugbee B. Koerner G. Albrechtsen R. Dewey W. Clawson S. (1999). ““USU-apogee“ Wheat - registration,” in *Dwarf Crops*, *Paper* , 11. Available at: https://digitalcommons.usu.edu/cpl_dwarfcrops/11. 10.2135/cropsci1997.0011183x003700020053x 11542531

[B4] Chen Y. Zhang L. Zhou Y. Geng Y. Chen Z. (2000). Inducing somatic meiosis-like reduction at high frequency by caffeine in root-tip cells of Vicia faba. Mutat. Res. - Fundam. Mol. Mech. Mutagenesis 452, 67–72. doi: 10.1016/S0027-5107(00)00045-2 10894892

[B5] Chupeau M. C. Granier F. Pichon O. Renou J. P. Gaudin V. Chupeaua Y. (2013). Characterization of the early events leading to totipotency in an arabidopsis protoplast liquid culture by temporal transcript profiling. Plant Cell 25, 2444–2463. doi: 10.1105/tpc.113.109538 23903317 PMC3753376

[B6] Clough S. J. Bent A. F. (1998). Floral dip: a simplified method for Agrobacterium -mediated transformation of Arabidopsis thaliana. Plant J. 16, 735–743. doi: 10.1046/j.1365-313x.1998.00343.x 10069079

[B7] Cook T. M. Biswas E. Dutta S. Aboobucker S. I. Hazinia S. Lübberstedt T. (2024). Assessing data analysis techniques in a high-throughput meiosis-like induction detection system. Plant Methods 20, 7. doi: 10.1186/s13007-023-01132-9 38212773 PMC10785433

[B8] Cook T. M. Isenegger D. Dutta S. Sahab S. Kay P. Aboobucker S. I.. (2023). Overcoming roadblocks for in *vitro* nurseries in plants: induction of meiosis. Front. Plant Sci. 14. doi: 10.3389/fpls.2023.1204813 PMC10272530 37332695

[B9] Davey M. R. Anthony P. Power J. B. Lowe K. C. (2005). Plant protoplasts: Status and biotechnological perspectives. Biotechnol. Adv. 23, 131–171. doi: 10.1016/j.biotechadv.2004.09.008 15694124

[B10] Daxinger L. Hunter B. Sheikh M. Jauvion V. Gasciolli V. Vaucheret H.. (2008). Unexpected silencing effects from T-DNA tags in Arabidopsis. Trends Plant Sci. 13, 4–6. doi: 10.1016/j.tplants.2007.10.007 18178509

[B11] De La Fuente G. N. Frei U. K. Lübberstedt T. (2013). Accelerating plant breeding. Trends Plant Sci. 18, 667–672. doi: 10.1016/j.tplants.2013.09.001 24080381

[B12] Eeckhaut T. Lakshmanan P. S. Deryckere D. Van Bockstaele E. Van Huylenbroeck J. (2013). Progress in plant protoplast research. Planta 238, 991–1003. doi: 10.1007/s00425-013-1936-7 23955146

[B13] Espinosa M.E.Á. Moreira R. O. Lima A. A. Ságio S. A. Barreto H. G. Luiz S. L. P.. (2017). Early histological, hormonal, and molecular changes during pineapple (Ananas comosus (L.) Merrill) artificial flowering induction. J. Plant Physiol., 209, 11–19. doi: 10.1016/j.jplph.2016.11.009 27988471

[B14] Fink D. Wohrer S. Pfeffer M. Tombe T. Ong C. J. Sorensen P. H. B. (2010). Ubiquitous expression of the monomeric red fluorescent protein mcherry in transgenic mice. Genesis 48, 723–729. doi: 10.1002/dvg.20677 20853428

[B15] Ghosh S. Watson A. Gonzalez-Navarro O. E. Ramirez-Gonzalez R. H. Yanes L. Mendoza-Suárez M.. (2018). Speed breeding in growth chambers and glasshouses for crop breeding and model plant research. Nat. Protoc. 13, 2944–2963. doi: 10.1038/s41596-018-0072-z 30446746

[B16] Hackett W. P. (1985). “Juvenility, maturation, and rejuvenation in woody plants,” in Horticultural reviews, ed. Janick J.. 109–155. doi: 10.1002/9781118060735.ch3

[B17] Holsters M. de Waele D. Depicker A. Messens E. van Montagu M. Schell J. (1978). Transfection and transformation of Agrobacterium tumefaciens. Mol. Gen. Genet. MGG 163, 181–187. doi: 10.1007/BF00267408 355847

[B18] Jähne F. Hahn V. Würschum T. Leiser W. L. (2020). Speed breeding short-day crops by LED-controlled light schemes. Theor. Appl. Genet. 133, 2335–2342. doi: 10.1007/s00122-020-03601-4 32399653 PMC7360641

[B19] Karunakaran R. Mauchline T. H. Hosie A. H. F. Poole P. S. (2005). A family of promoter probe vectors incorporating autofluorescent and chromogenic reporter proteins for studying gene expression in Gram-negative bacteria. Microbiology 151, 3249–3256. doi: 10.1099/mic.0.28311-0 16207908

[B20] Li G. Boontung R. Powers C. Belamkar V. Huang T. Miao F.. (2017). Genetic basis of the very short life cycle of ‘Apogee’ wheat. BMC Genomics 18, 838. doi: 10.1186/s12864-017-4239-8 29089022 PMC5664786

[B21] Li H. Rasheed A. Hickey L. T. He Z. (2018). Fast-forwarding genetic gain. Trends Plant Sci. 23, 184–186. doi: 10.1016/j.tplants.2018.01.007 29426713

[B22] Lübberstedt T. Frei U. K. (2012). Application of doubled haploids for target gene fixation in backcross programmes of maize. Plant Breed. 131, 449–452. doi: 10.1111/j.1439-0523.2011.01948.x

[B23] Matzke M. A. Primig M. Trnovsky J. Matzke A. J. M. (1989). Reversible methylation and inactivation of marker genes in sequentially transformed tobacco plants. EMBO J. 8, 643–649. doi: 10.1002/embj.1989.8.issue-3 16453872 PMC400855

[B24] McCaw M. E. Wallace J. G. Albert P. S. Buckler E. S. Birchler J. A. (2016). Fast-flowering mini-maize: Seed to seed in 60 days. Genetics 204, 35 LP–35 42. doi: 10.1534/genetics.116.191726 27440866 PMC5012399

[B25] Medrano J. V. Martínez-Arroyo A. M. Míguez J. M. Moreno I. Martínez S. Quiñonero A.. (2016). Human somatic cells subjected to genetic induction with six germ line-related factors display meiotic germ cell-like features. Sci. Rep. 6, 24956. doi: 10.1038/srep24956 27112843 PMC4844986

[B26] Mieulet D. Jolivet S. Rivard M. Cromer L. Vernet A. Mayonove P.. (2016). Turning rice meiosis into mitosis. Cell Res. 26, 1242–1254. doi: 10.1038/cr.2016.117 27767093 PMC5099866

[B27] Mlotshwa S. Pruss G. J. Gao Z. Mgutshini N. L. Li J. Chen X.. (2010). Transcriptional silencing induced by Arabidopsis T-DNA mutants is associated with 35S promoter siRNAs and requires genes involved in siRNA-mediated chromatin silencing. Plant J. 64, 699–704. doi: 10.1111/tpj.2010.64.issue-4 21070421 PMC3059090

[B28] Murray S. C. Eckhoff P. Wood L. Paterson A. H. (2013). A proposal to use gamete cycling in *vitro* to improve crops and livestock. Nat. Biotechnol. 31, 877–880. doi: 10.1038/nbt.2707 24104748

[B29] Nelms B. Walbot V. (2019). Defining the developmental program leading to meiosis in maize. Science 364, 52 LP–52 56. doi: 10.1126/science.aav6428 30948545

[B30] Pandey S. Singh A. Parida S. K. Prasad M. (2022). Combining speed breeding with traditional and genomics-assisted breeding for crop improvement. Plant Breed. 141, 301–313. doi: 10.1111/pbr.13012

[B31] Peterson R. A. (2021). Finding Optimal Normalizing Transformations via bestNormalize. R J. 13, 310–329. doi: 10.32614/RJ-2021-041

[B32] Rodrmguez E. P. B. Morante N. Salazar S. Hyde P. T. Setter T. L. Kulakow P.. (2023). Flower-inducing technology facilitates speed breeding in cassava. Front. Plant Sci. 14. doi: 10.3389/fpls.2023.1172056 PMC10239864 37284728

[B33] Schubert D. Lechtenberg B. Forsbach A. Gils M. Bahadur S. Schmidt R. (2004). Silencing in arabidopsis T-DNA transformants: the predominant role of a gene-specific RNA sensing mechanism versus position effects. Plant Cell 16, 2561–2572. doi: 10.1105/tpc.104.024547 15367719 PMC520955

[B34] Shaner N. C. Campbell R. E. Steinbach P. A. Giepmans B. N. G. Palmer A. E. Tsien R. Y. (2004). Improved monomeric red, orange and yellow fluorescent proteins derived from Discosoma sp. red fluorescent protein. Nat. Biotechnol. 22, 1567–1572. doi: 10.1038/nbt1037 15558047

[B35] Sheen J. (2001). Signal transduction in maize and Arabidopsis mesophyll protoplasts. Plant Physiol. 127, 1466–1475. doi: 10.1104/pp.010820 11743090 PMC1540179

[B36] Vickers C. E. Schenk P. M. Li D. Mullineaux P. M. Gresshoff P. M. (2007). pGFPGUSPlus, a new binary vector for gene expression studies and optimising transformation systems in plants. Biotechnol. Lett. 29, 1793–1796. doi: 10.1007/s10529-007-9467-6 17687623

[B37] Watson A. Ghosh S. Williams M. J. Cuddy W. S. Simmonds J. Rey M.-D.. (2018). Speed breeding is a powerful tool to accelerate crop research and breeding. Nat. Plants 4, 23–29. doi: 10.1038/s41477-017-0083-8 29292376

[B38] Yan G. Liu H. Wang H. Lu Z. Wang Y. Mullan D.. (2017). Accelerated generation of selfed pure line plants for gene identification and crop breeding. Front. Plant Sci. 8. doi: 10.3389/fpls.2017.01786 PMC5660708 29114254

[B39] Yihua C. Lihua Z. Yuxuan G. Zhenghua C. (2001). Meiosis-Like Reduction during Somatic Embryogenesis of Arabidopsis thaliana. *In Vitro* Cellular & Developmental Biology. Plant 37, 654–657.

[B40] Yoo S.-D. Cho Y.-H. Sheen J. (2007). Arabidopsis mesophyll protoplasts: a versatile cell system for transient gene expression analysis. Nat. Protoc. 2, 1565. doi: 10.1038/nprot.2007.199 17585298

[B41] Yoshida H. Yamaguchi H. (1973). Arrangement and association of somatic chromosomes induced by chloramphenicol in barley. Chromosoma 43, 399–407. doi: 10.1007/BF00406746 4776472

