# Supplementary material for: A cell-based fluorescent system and statistical framework to detect meiosis-like induction in plants
Source: Front Plant Sci. 2024 Jul 8;15:1386274. doi: 10.3389/fpls.2024.1386274 (PMC11260738; doi:10.3389/fpls.2024.1386274)
Supplement: Supplementary Table 3 — Chi-squared results for determining if RFP and GFP are present in single-loci. [file Table_3.pdf]

Genotype	O and E	Resistant	Susceptible	No. of plants	Chi-Squared value	P-value	Chi-Squared Critical value (alpha=0.05)
GFP	Expected	52.5	17.5	70	2.3	0.13	3.841
	Observed	58	12				
RFP	Expected	52.5	17.5	70	0.93	0.33	3.841
	Observed	56	14				
