# Supplementary figures and images for: A cell-based fluorescent system and statistical framework to detect meiosis-like induction in plants
Source: Front Plant Sci. 2024 Jul 8;15:1386274. doi: 10.3389/fpls.2024.1386274 (PMC11260738; doi:10.3389/fpls.2024.1386274)

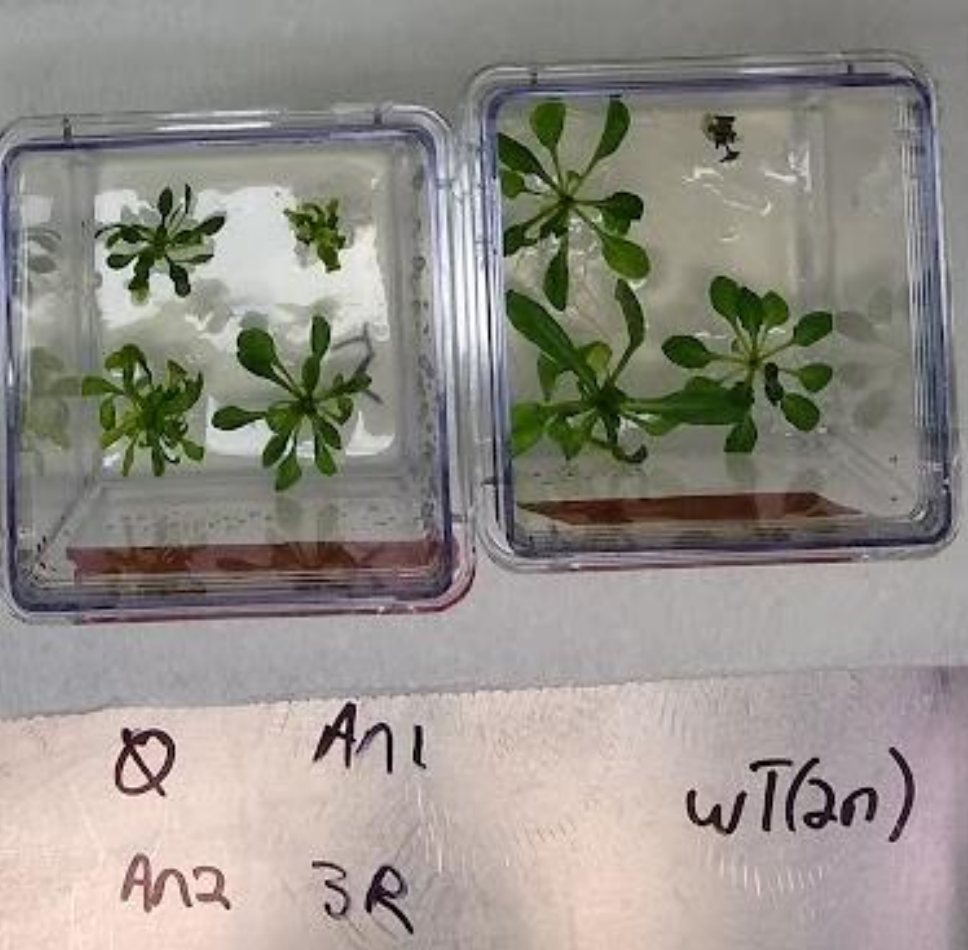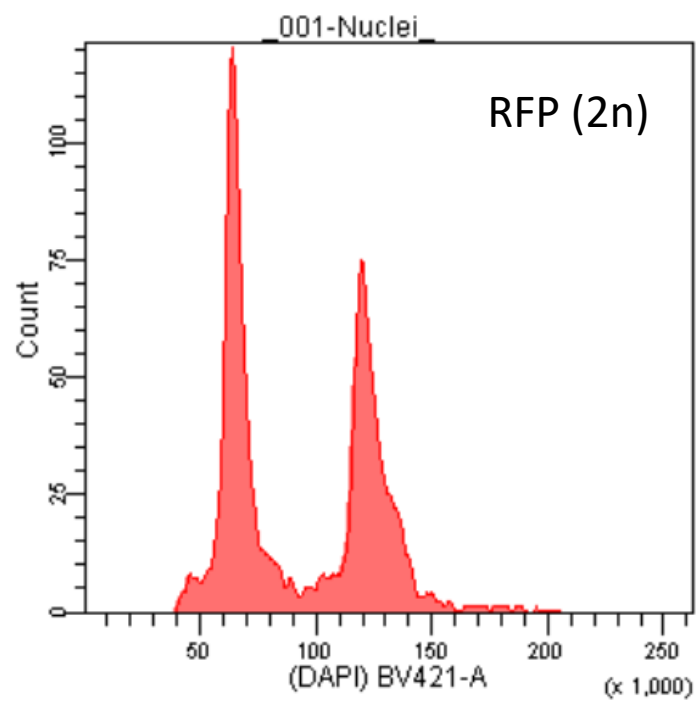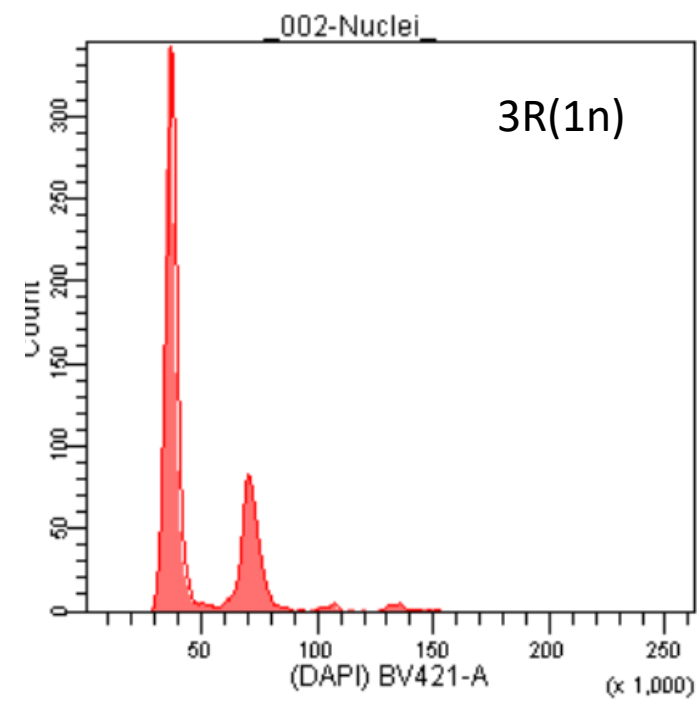

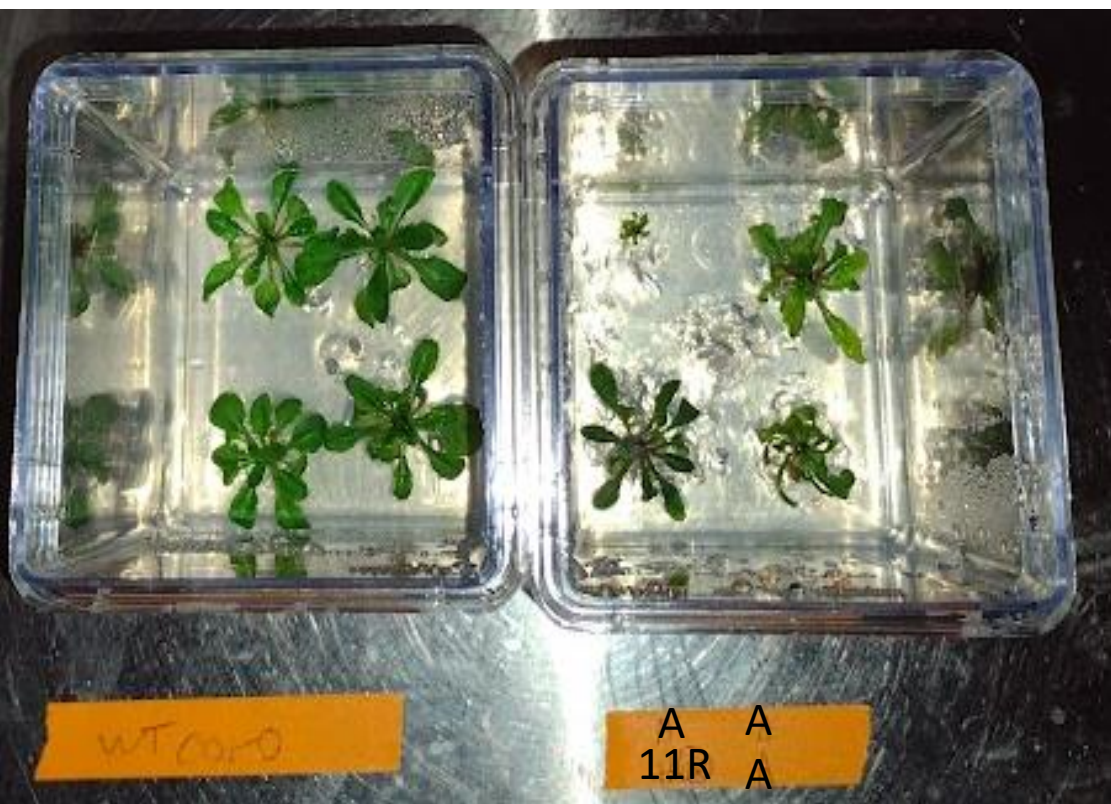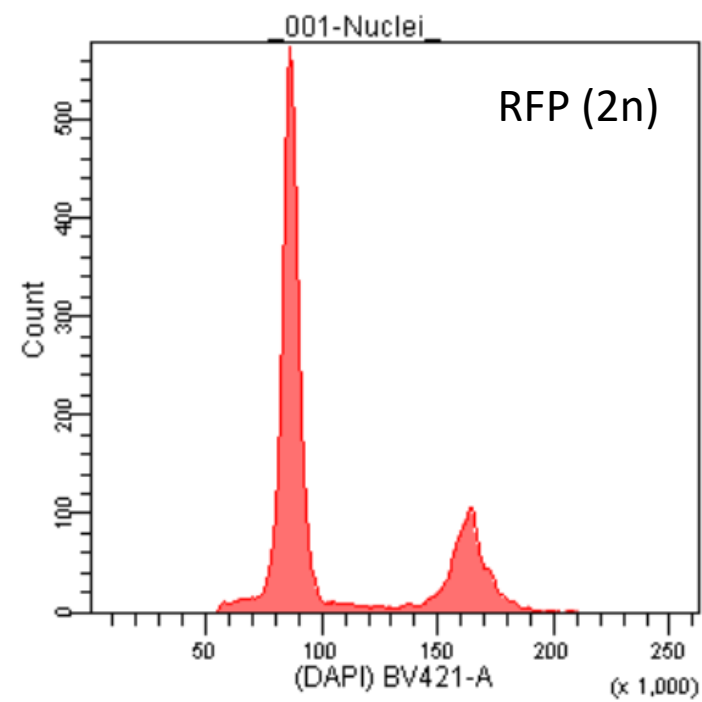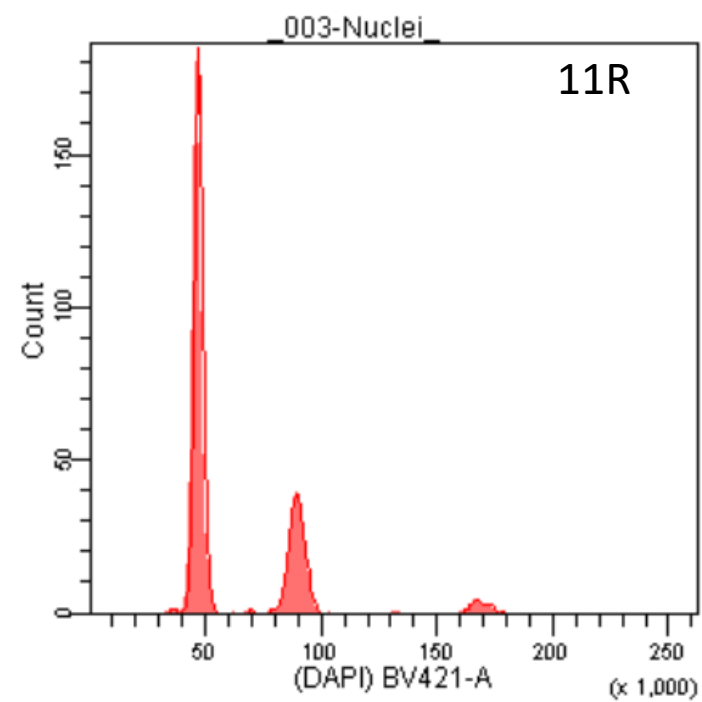

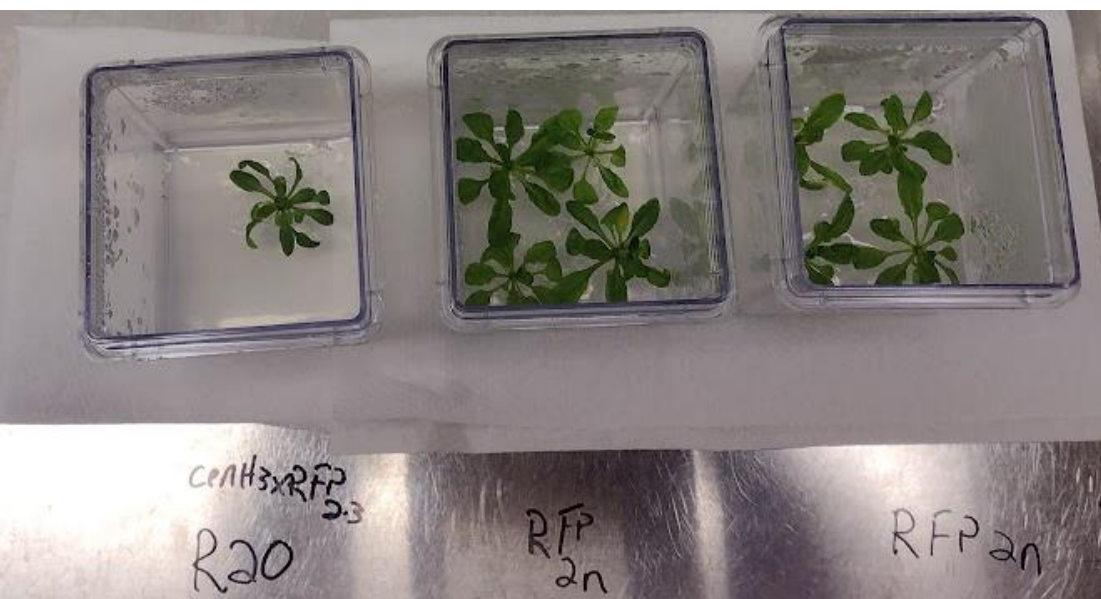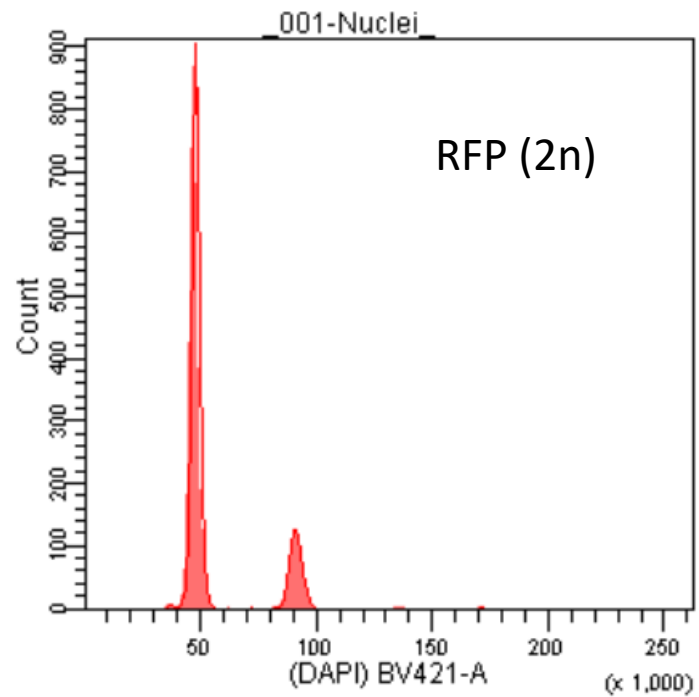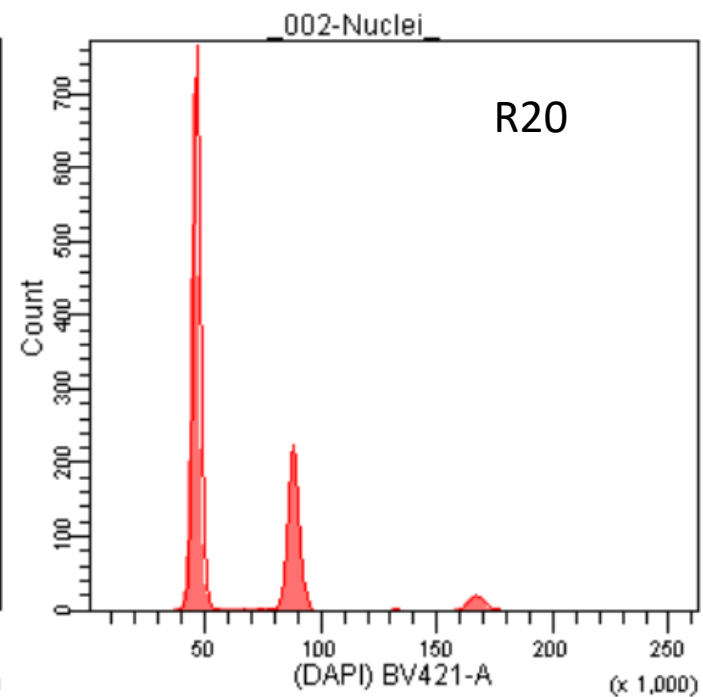

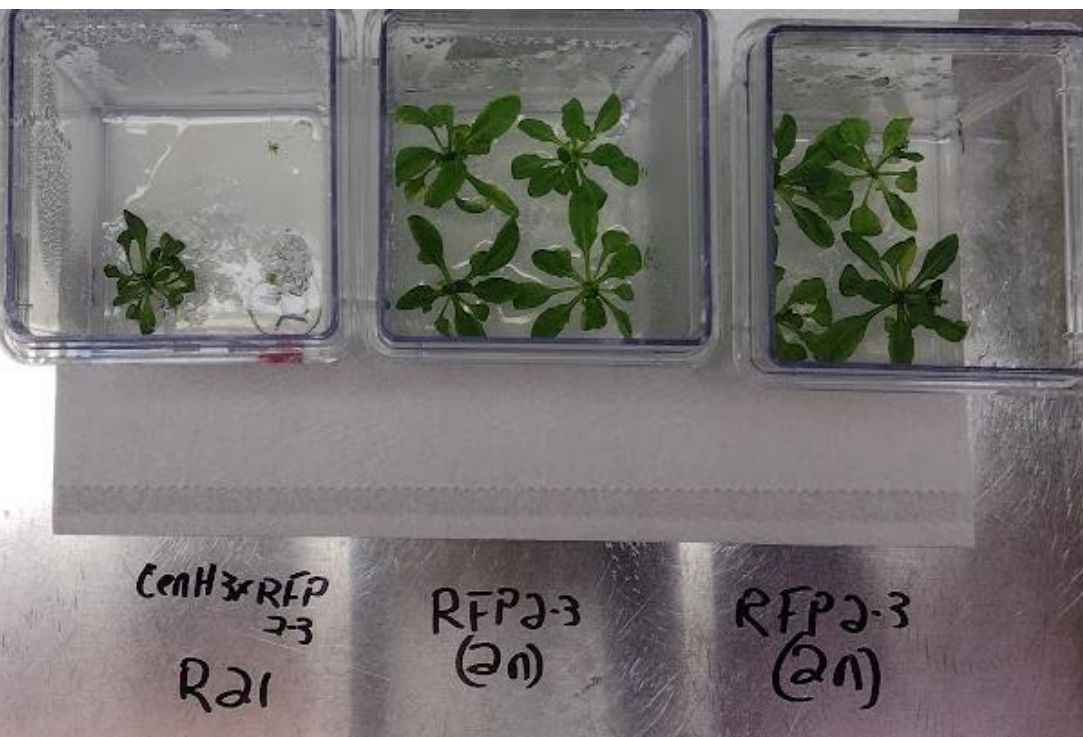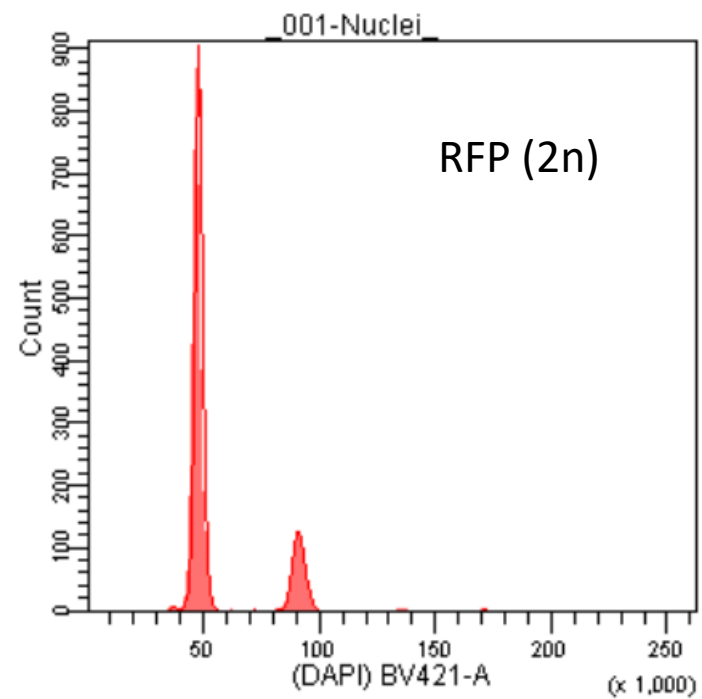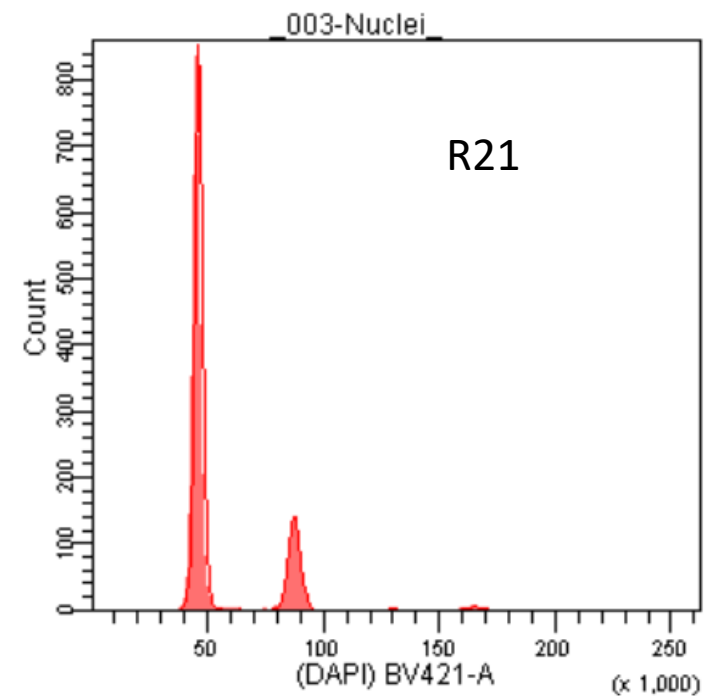

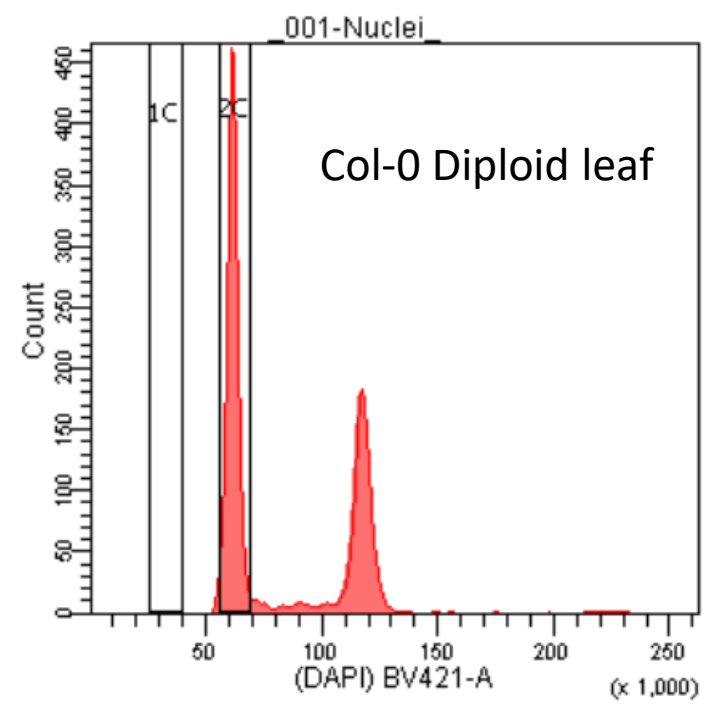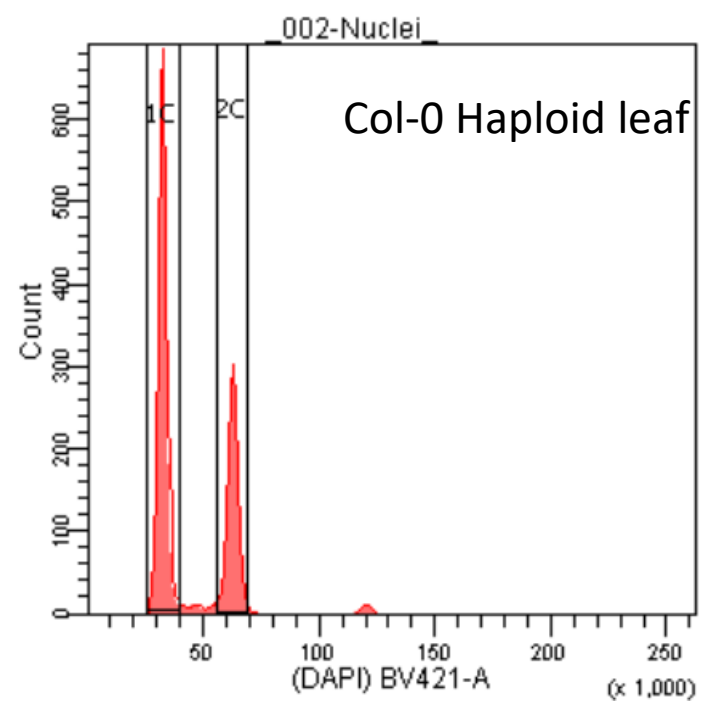

Supplement: Supplementary Figure 1 — Plant phenotyping and flow cytometry data for haploid or diploid determination. [file DataSheet_1.pdf]
