# Supplementary material for: A cell-based fluorescent system and statistical framework to detect meiosis-like induction in plants
Source: Front Plant Sci. 2024 Jul 8;15:1386274. doi: 10.3389/fpls.2024.1386274 (PMC11260738; doi:10.3389/fpls.2024.1386274)
Supplement: Supplementary Figure 2 — Root and protoplast screening results of two high-expressing RFP lines. [file DataSheet_2.pdf]

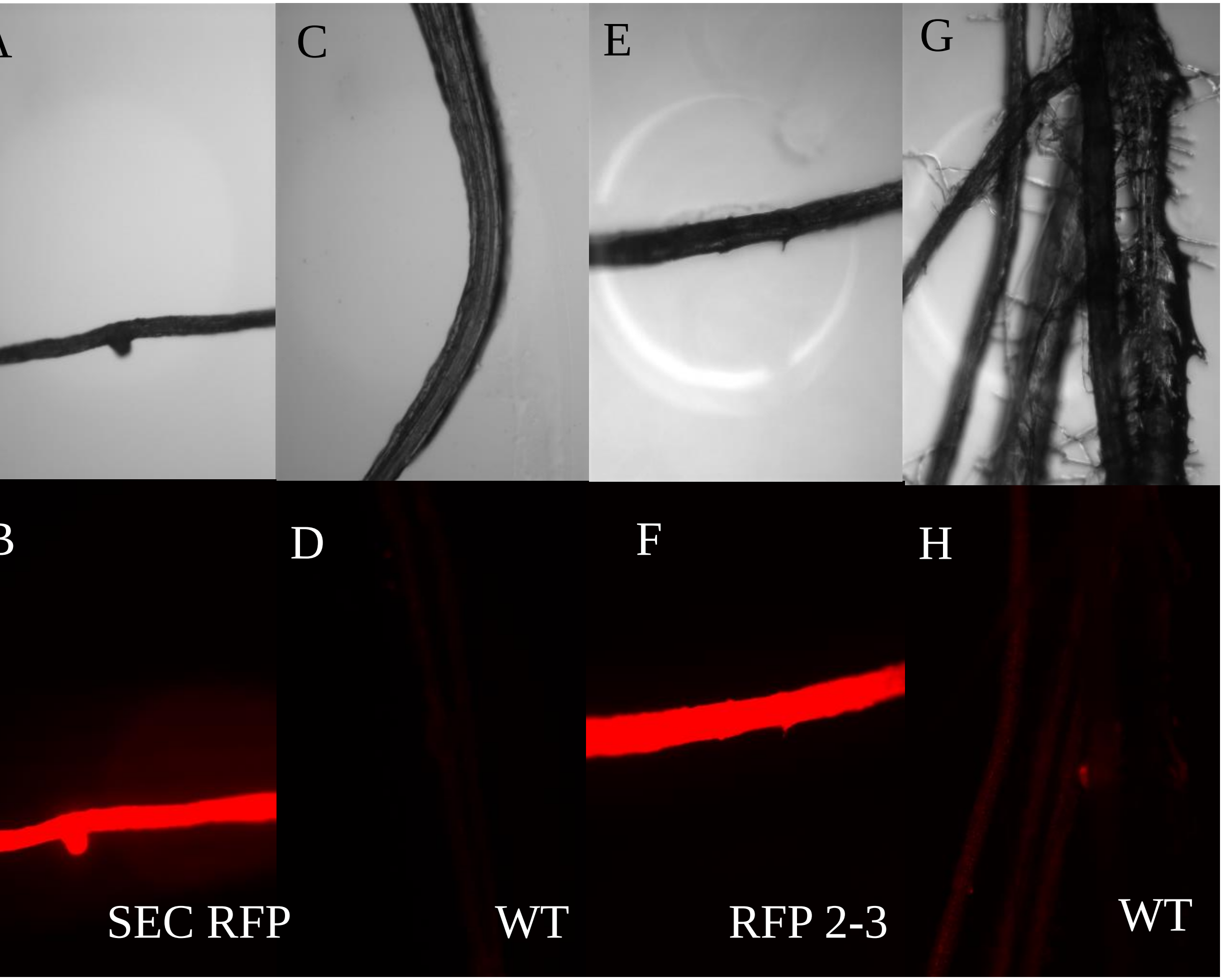

I

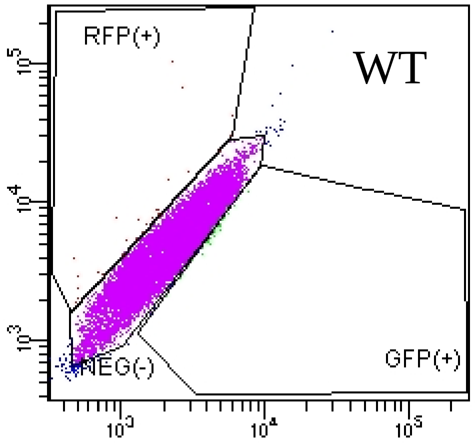

Classification	Events	% Parent
Protoplasts	10,190	99.8
Neg	9,957	97.7
GFP	83	0.8
RFP	34	0.3

J

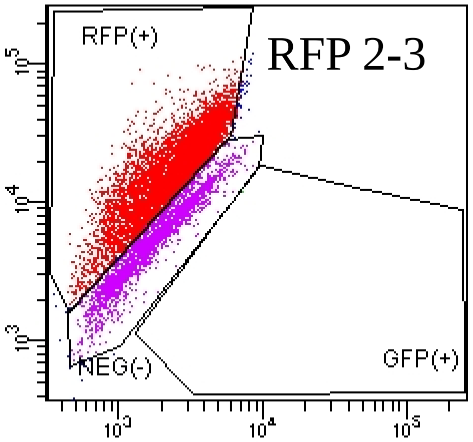

Classification	Events	% Parent
Protoplasts	10,483	99.9
Neg	2,695	25.7
GFP	1	<0.1
RFP	7,718	73.6

K

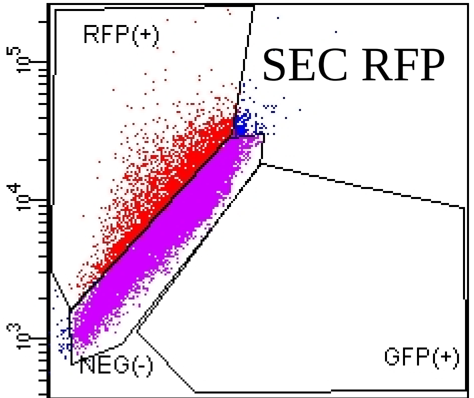

Classification	Events	% Parent
Protoplasts	13,768	99.6
Neg	10,475	76.1
GFP	0	0
RFP	3,006	21.8
