# Supplementary material for: A cell-based fluorescent system and statistical framework to detect meiosis-like induction in plants
Source: Front Plant Sci. 2024 Jul 8;15:1386274. doi: 10.3389/fpls.2024.1386274 (PMC11260738; doi:10.3389/fpls.2024.1386274)
Supplement: Supplementary Figure 3 — Root and protoplast screening results of two high-expressing GFP lines. [file DataSheet_3.pdf]

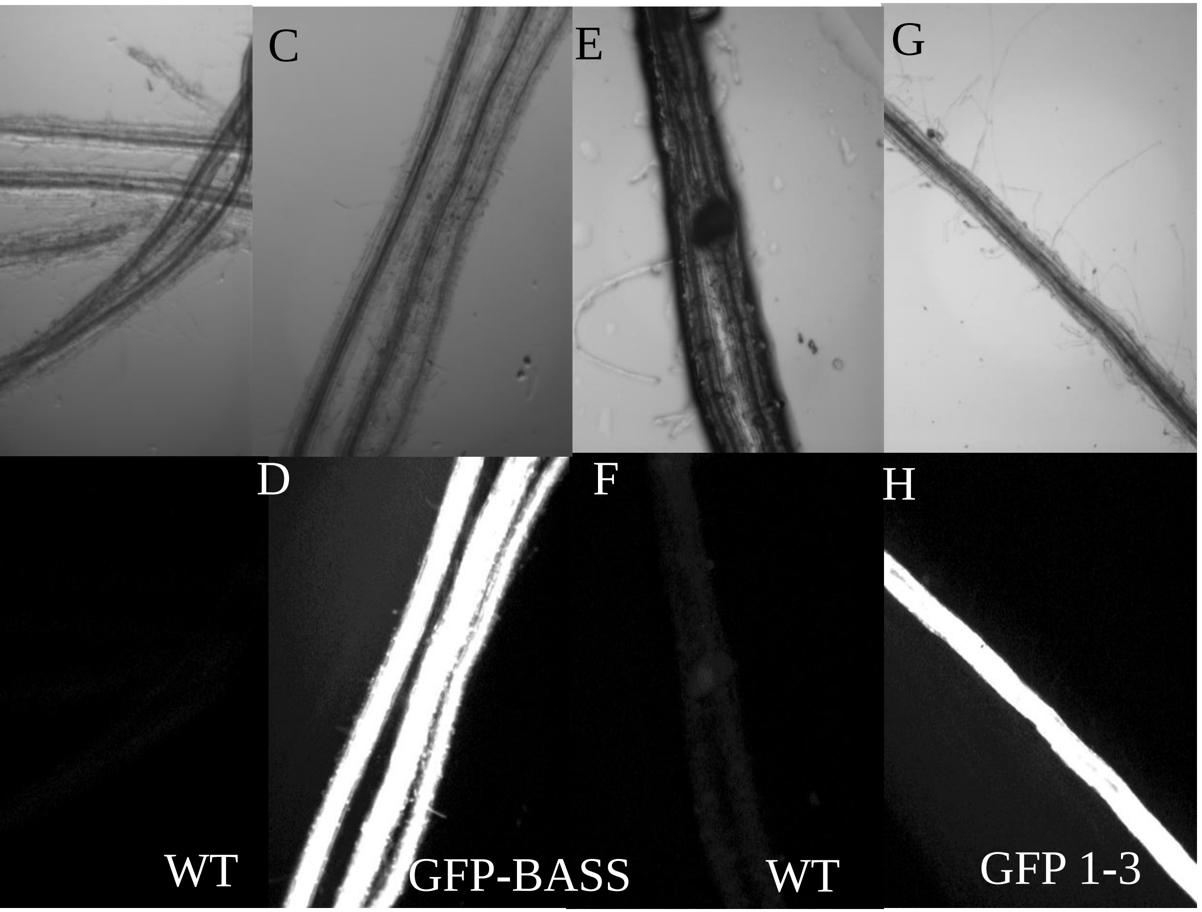

I

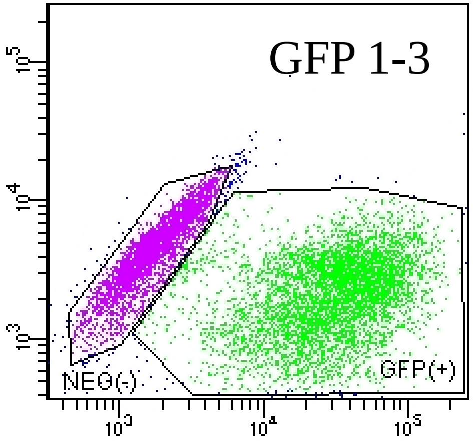

Classification	Events	% Parent
Protoplasts	9,361	99.2
Neg	3,530	37.7
GFP	5,374	57.4

J

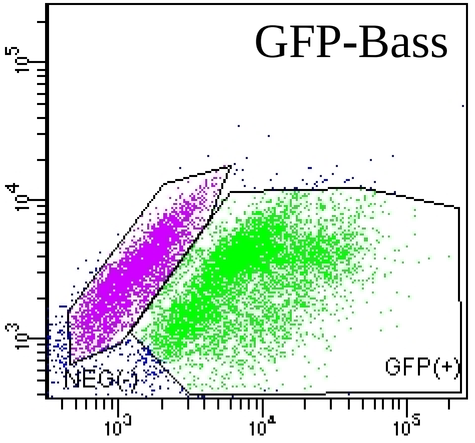

Classification	Events	% Parent
Protoplasts	9,535	98.2
Neg	3,392	35.6
GFP	5,657	59.3

K

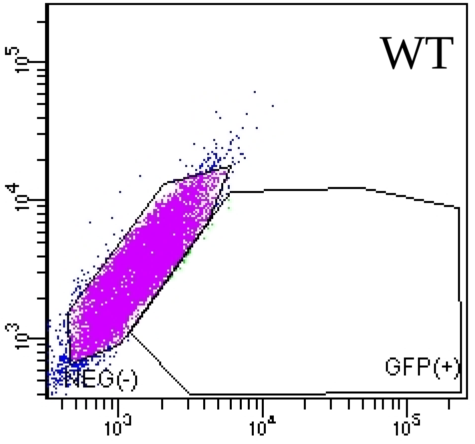

Classification	Events	% Parent
Protoplasts	9,273	99.0
Neg	8,776	94.6
GFP	17	0.2
